# ERK-mediated phosphorylation modulates the autolytic processing of the cysteine protease CAPN3

**DOI:** 10.1016/j.jbc.2026.113638

**Published:** 2026-10-14

**Authors:** Chihiro Hisatsune, Fumiko Shinkai-Ouchi, Mayumi Shindo, Shoji Hata, Yasuko Ono

**Affiliations:** 1 Calpain Group, Tokyo Metropolitan Institute of Medical Science, Tokyo, Japan; 2 Protein Analysis, Center for Basic Technology Research, Tokyo Metropolitan Institute of Medical Science, Tokyo, Japan; 3 Integrated Research Administration Center, Saitama Medical University, Saitama, Japan

**Keywords:** calpain, ERK, muscular dystrophy, phosphorylation

## Abstract

Calpain-3 (CAPN3) is a Ca^2+^-dependent cysteine protease predominantly expressed in skeletal muscles. Mutations in CAPN3 are associated with limb-girdle muscular dystrophy R1 (LGMDR1), an autosomal recessive muscular dystrophy. CAPN3 is uniquely activated by rapid autolytic processing; however, the mechanisms regulating this activation are poorly understood. Here we show that phosphorylation regulates CAPN3 autolytic processing in living cells. We found that ouabain, a cardiac glycoside, activated ERK, which induced the phosphorylation of CAPN3 at S19 located within its N-terminal region. This phosphorylation promoted the N-terminal cleavage without altering the autolytic insertion sequence 1 (IS1) processing of CAPN3 or its subcellular localization in skeletal muscle cells. Notably, treatment of cultured skeletal muscle cells with insulin-like growth factor I (IGF-1), which can activate the ERK pathway, also induced S19 phosphorylation, suggesting that this modification can occur downstream of physiologically relevant extracellular signaling. These results demonstrate that ERK-mediated phosphorylation provides a regulatory mechanism for modulating N-terminal processing of CAPN3 without substantially affecting IS1 autolytic processing.

Calpains are Ca^2+^-dependent cysteine proteases that regulate diverse biological processes, including cytoskeletal remodeling, cell differentiation, migration, and apoptosis by modulating proteins through limited proteolysis 1, 2, 3. Among the 15 human calpains identified to date, calpain-3 (CAPN3/p94) is a classical calpain that is predominantly expressed in skeletal muscle 4, 5. Notably, mutations in the *CAPN3* gene cause limb-girdle muscular dystrophy R1 (LGMDR1) (6), which has prompted many researchers to investigate the activation mechanisms and physiological functions of CAPN3 in skeletal muscle.

The Ca^2+^-dependent activation of the calpain family has been extensively studied in the ubiquitously expressed classical members, calpain-1 and calpain-2. Both exist as heterodimers, comprising an 80-kDa large catalytic subunit and a 30-kDa regulatory subunit (7). The catalytic subunit contains four distinct domains: an N-terminal anchor helix, a protease core (PC1 and PC2), a calpain-type β-sandwich (CBSW) domain, and a penta-EF (PEF) domain, while the regulatory subunit consists of a glycine-rich domain and PEF domain (8). In the absence of Ca^2+^, calpain-2 forms a flat oval disc-like structure that is stabilized by two major intermolecular interfaces: one between the PEF domains of the large and small subunits, and the other between the N-terminal anchor helix and the PEF domain of the large and small subunits, respectively 9, 10. Because such positioning of the N-terminal anchor helix restricts the assembly of the catalytic triad residues in the catalytic subunit, the enzyme is inactive in the absence of Ca^2+^. Upon Ca^2+^ binding, which occurs cooperatively at two sites within the protease core and the regulatory PEF domain, the constraint imposed by the helix is relieved, leading to the formation of a functional catalytic cleft in the protease domain and subsequent enzyme activation 11, 12.

CAPN3, which shares the fundamental architecture of the classical calpains described earlier, contains three additional unique insertion sequences: an N-terminal sequence (NS), an insertion sequence 1 (IS1) within PC2, and an insertion sequence 2 (IS2) between the CBSW and PEF domains (4) (Fig. 1*A*). The autolytic removal of the IS1 region is essential for CAPN3 to exert its full enzymatic activity 13, 14. Unlike CAPN1 and CAPN2, which require a significant elevation in the cytosolic calcium concentration for activation, CAPN3 undergoes spontaneous activation in heterologous cells, even without extracellular stimuli (13). This rapid activation hinders structural studies aimed at resolving the precise mechanism of Ca^2+^-dependent activation of the full-length protein; consequently, the precise regulatory mechanisms that regulate CAPN3 autolysis in living cells remain poorly understood (8).

**Figure 1:**
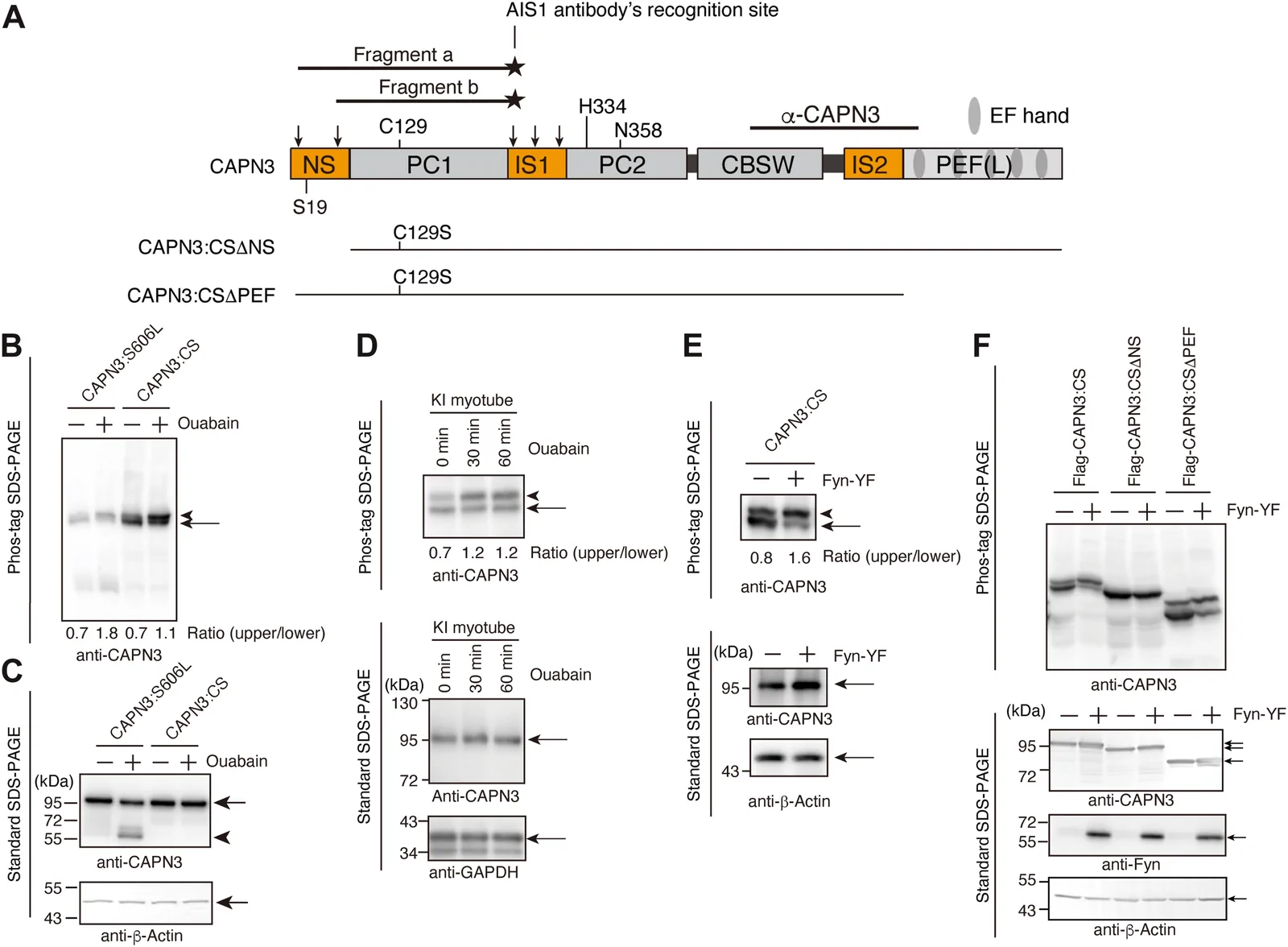
**Ouabain enhances CAPN3 phosphorylation.***A*, Schematic representation of CAPN3 and its deletion mutants. *Arrows* indicate the autolytic cleavage sites within the NS and IS1 regions of CAPN3. Fragments a and b are the autolytic products recognized by an anti-AIS1 antibody. Residues C129, H334, and N358 constitute the catalytic center, while S19 is the phosphorylation site identified in this study. *B*, Ouabain induces the phosphorylation of CAPN3:S606L and CAPN3:CS in HeLa cells. Cell lysates from transfected cells treated with 1 mM ouabain for 60 min were analyzed by western blotting with an anti-CAPN3 antibody on a Phos-tag SDS-PAGE. The *upper arrowhead* marks the mobility-shifted (phosphorylated) CAPN3, whereas the *lower arrow* indicates the non-phosphorylated form. N = 4. *C*, Western blot of ouabain-treated cell lysates expressing CAPN3:S606L and CAPN3:CS on a standard SDS-PAGE. The membrane was probed with anti-CAPN3 (*upper panel*) and anti-β-Actin antibody (*lower panel*). Arrowhead indicates autolytic ∼55 kDa band of CAPN3:S606L. N = 3. *D*, Ouabain increases the relative abundance of phosphorylated CAPN3:CS in cultured skeletal myotubes derived from CAPN3KI mice. Differentiated cells (7 days) were treated with 1 mM ouabain for 60 min. The *arrowhead* and the *arrow* in the *upper panel* indicate the phosphorylated and non-phosphorylated forms of CAPN3:CS on a Phos-tag gel, respectively. N = 3. The l*ower panel* shows western blotting of the same samples on standard SDS-PAGE, with GAPDH as the loading control. *E*, co-expression of the constitutively active Fyn-YF increases the phosphorylation of CAPN3:CS in HeLa cells. The *upper* and the *lower panels* indicate Western blots on Phos-tag SDS-PAGE and a standard SDS-PAGE gels, respectively. N = 3. *F*, Fyn-YF increases the phosphorylated band of CAPN3:CS:ΔPEF, but not that of CAPN3:CS:ΔNS in HEK293T cells. The deletion mutants are depicted in Figure 1*A*. N = 3. The *upper* and the *lower panels* indicate Western blot on a Phos-tag SDS-PAGE and a standard SDS-PAGE, respectively.

We recently reported that ouabain, a cardiac glycoside, is an ideal chemical compound for triggering CAPN3 activation by inducing a small but sustained increase in cytosolic Ca^2+^ levels in heterologous cells and cultured skeletal myotubes (15). Ouabain also activates multiple signaling pathways in addition to Ca^2+^ mobilization (16), raising the possibility that these pathways modulate CAPN3 through additional post-translational mechanisms. Because phosphorylation regulates several other members of the calpain family 17, 18, 19, 20, 21, 22, 23, we investigated whether signaling through the Ras/Raf/ERK pathway induces CAPN3 phosphorylation and affects its processing in living cells.

## Results

### Ouabain increases the phosphorylation of CAPN3 in cultured skeletal myotubes

To investigate the potential mechanism by which ouabain modulates the autolytic processing of CAPN3, we focused on CAPN3 phosphorylation, as ouabain is known to trigger various signaling pathways such as the phospholipase C/IP_3_ receptor/Ca^2+^ signaling, activation of tyrosine kinases, and Ras-Raf-ERK signaling (16).

Because of the intrinsic instability of wild-type (WT) CAPN3 due to its rapid autolysis when expressed in heterologous cells, we used stable and high-expressing CAPN3 mutants with abolished or reduced protease activity to enable clear detection of CAPN3 phosphorylation. A protease-dead mutant of CAPN3 (CAPN3:CS) and an LGMDR1-associated mutant with reduced activity (CAPN3:S606L) were expressed in HeLa cells, and the cells were stimulated with ouabain. The cell lysates were loaded onto a Phos-tag SDS-PAGE gel (Fig. 1*B*), which separates phosphorylated proteins based on their decreased mobility (24), as well as onto a standard SDS-PAGE gel (Fig. 1*C*), and they were analyzed by Western blotting. Before ouabain stimulation, we observed doublet bands of CAPN3 on the Phos-tag SDS-PAGE, with the lower band exhibiting a stronger signal than the mobility-shifted upper band (Fig. 1*B*). Following ouabain stimulation, we observed an increase in the signal intensity of the upper band in both CAPN3:S606L and CAPN3:CS (Fig. 1*B*), suggesting that the lower and upper bands corresponded to the non-phosphorylated and phosphorylated forms, respectively. On a standard SDS-PAGE gel, we observed a single band of CAPN3:CS in both the ouabain-treated and ouabain-untreated cells (Fig. 1*C*). As we previously reported 15, 25, ouabain induced autolysis of CAPN3:S606L, resulting in the appearance of a faster-migrating band at approximately 50 kDa. Nevertheless, no detectable mobility shift of the full-length CAPN3:S606L band was observed on a standard SDS-PAGE gel (Fig. 1*C*). These results suggested that CAPN3 was phosphorylated in response to ouabain stimulation.

To further validate the phosphorylation of CAPN3 in living cells, we utilized skeletal myotubes derived from CAPN3:CS knock-in mice (CAPN3KI) expressing a protease-inactive form of CAPN3 (26). As shown in Figure 1*D*, ouabain stimulation robustly increased the upper phosphorylated band of endogenous CAPN3:CS in a Phos-tag SDS-PAGE gel (Fig. 1*D*, upper panel), whereas such a mobility shift was not detected in a standard SDS-PAGE gel (Fig. 1*D*, lower panel), confirming that ouabain triggered CAPN3 phosphorylation in cultured skeletal myotubes.

Since ouabain is known to activate Src family tyrosine kinases leading to stimulation of downstream pathways such as MEK/ERK (16), we next co-expressed CAPN3:CS with a constitutively active form of Fyn, a member of the Src family tyrosine kinases (Fyn-YF) (27) in HeLa cells. Co-expression with Fyn-YF significantly increased the intensity of the phosphorylated CAPN3 band in a Phos-tag SDS-PAGE gel (Fig. 1*E*, upper panel), whereas no band shift was detected in a standard SDS-PAGE gel (Fig. 1*E*, lower panel). Since the band mobility shift observed with Fyn-YF co-expression was similar to that induced by ouabain stimulation, we reasoned that both stimuli might target the same amino acid residue(s) of CAPN3.

To map the phosphorylation region, we generated deletion mutants of CAPN3:CS lacking either the NH_2_-terminal sequence (CAPN3:CS:ΔNS) or the COOH-terminal PEF domain (CAPN3:CS:ΔPEF). We co-expressed these mutants with Fyn-YF in HEK293T cells, which abundantly express exogenous proteins, and examined their band shifts on the Phos-tag SDS-PAGE gel (Fig. 1*F*, upper panel) and on the standard SDS-PAGE gel (Fig. 1*F*, lower panel). While CAPN3:CS:ΔPEF retained the propensity for a mobility shift upon Fyn-YF co-expression, the Fyn-YF-dependent mobility shift was completely abolished in CAPN3:CS:ΔNS (Fig. 1*F*, upper panel). None of these mutants showed a detectable mobility shift in the presence or absence of Fyn on a standard SDS-PAGE gel (Fig. 1*F*, lower panel). These results strongly suggested that the NS region of CAPN3 contains a primary site for CAPN3 phosphorylation.

### Extracellular signal-regulated kinase (ERK) phosphorylates CAPN3 at S19

To accurately determine the specific phosphorylated residues of CAPN3 upon Fyn-YF co-expression, mass spectrometry measurement was employed. We co-expressed maltose-binding protein (MBP)-tagged CAPN3:CS with or without Fyn-YF in HEK293T cells, purified the MBP-CAPN3:CS, and compared the phosphorylated residues in these two preparations. We identified various phosphorylated peptides, revealing the phosphorylation of multiple CAPN3 residues (Fig. 2*A* and Table S1). Among these, the peptides containing phospho-S19, which localizes within the NH_2_-terminal sequence of CAPN3, were detected at the highest frequency, irrespective of co-expression with Fyn-YF. We further compared the relative abundance of phosphorylated peptides corresponding to each phosphorylation site with and without the co-expression of Fyn-YF. The percentage of peptides containing phosphorylated serine 19 (S19) in CAPN3 increased significantly when MBP-CAPN3 was co-expressed with Fyn-YF (Fig. 2*B*).

**Figure 2:**
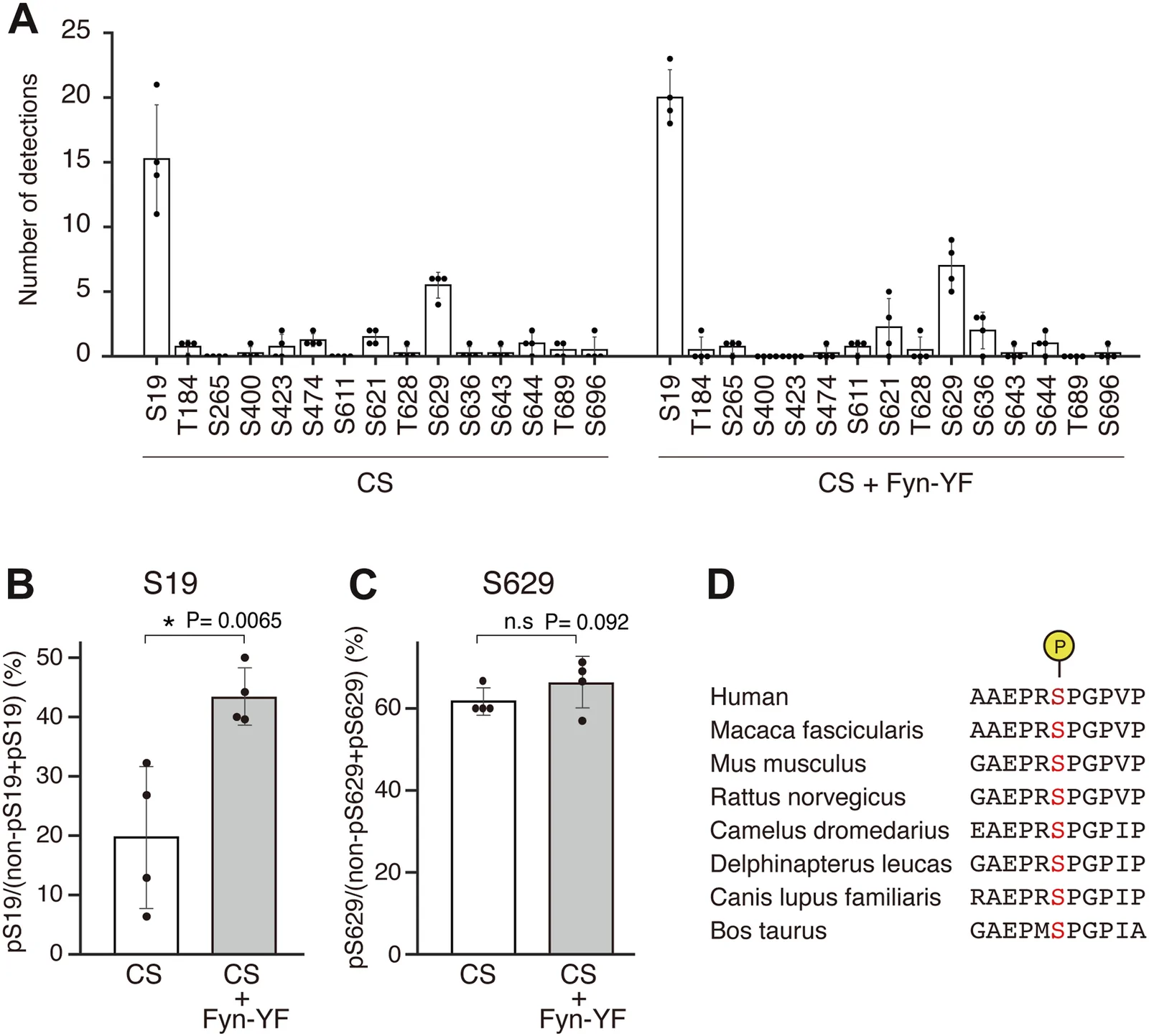
**Determination of phosphorylated residues in CAPN3.***A*, number of distinct phosphorylated peptides detected for CAPN3 with and without Fyn-YF co-expression. Two independent experiments were performed, with two technical replicate measurements per experiment (N = 4 measurements). *B*, ratio of phosphorylated to non-phosphorylated S19 peptides in the presence and absence of Fyn-YF co-expression. Fyn YF (−): 21.57 ± 9.56; Fyn-YF (+): 43.45 ± 4.84 (mean ± SD). ⁎*p* = 0.0065, *Two tailed unpaired Student’s t* test. *C*, ratio of phosphorylated to non-phosphorylated S629 peptides in the presence and absence of Fyn-YF co-expression. Fyn-YF (−): 58.33 ± 3.33; Fyn-YF (+): 66.37 ± 7.30 (mean ± SD). *p* = 0.092, *Two tailed unpaired Student’s t* test. *D*, multiple sequence alignment of CAPN3 from diverse species, highlighting the conservation of serine 19 across all analyzed sequences.

We previously reported CAPN3 phosphorylation at S629 and S636 in heterologous cells (23). In line with this, peptides containing phospho-S629 and phospho-S636 were also detected, although not frequently, in the present study; Fyn-YF co-expression was irrelevant to the phosphorylation state of these sites (Fig. 2*A*). For example, the relative abundance of peptides containing phosphorylated S629 (∼60%) slightly increased by Fyn-YF co-expression but not significantly (Fig. 2*C*). Nevertheless, the value, 60%, itself suggests the constant phosphorylation at S629 of CAPN3 expressed in HEK293T cells. On the other hand, the relative abundance of phospho-S636 was 3.4% even in the presence of Fyn-YF co-expression (Table S1), therefore, we reasoned that S636 was a less favored phosphorylation site in CAPN3 expressed in HEK293T cells.

Notably, the S19 residue is highly conserved among various mammals, including mice and rats (Fig. 2*D*), whereas residues S629 and S636 are not conserved in mice and rats, respectively (23). The high conservation of S19 suggests that phosphorylation at this site may have a conserved regulatory role in CAPN3.

### ERK phosphorylates CAPN3 at S19 in living cells

ERK is a well-known downstream signaling molecule of the Src family of tyrosine kinases, and the sequence surrounding S19 of CAPN3 matches an ERK phosphorylation consensus motif (P-X-S/T-P) (28). Therefore, we treated cells expressing CAPN3 mutants (Fig. 1*B*) with the ERK inhibitor SCH772984 prior to ouabain stimulation in order to examine the involvement of ERK in the phosphorylation of CAPN3. We found that the ERK inhibitor decreased the ouabain-induced mobility shift of the CAPN3:S606L band on a Phos-tag SDS-PAGE gel (Fig. 3*A*). No mobility shift was observed in the full length of S606L mutant in any of these conditions on a standard SDS-PAGE gel (Fig. 3*A*, arrow in the right upper panel). We also found that a delay in the migration of CAPN3:CS caused by the co-expression of Fyn-YF on Phos-tag SDS-PAGE was an ERK-dependent event (Fig. 3*B*, left panel). The ERK inhibitor treatment did not produce a detectable mobility shift of CAPN3:CS on a standard SDS-PAGE (Fig. 3*B*, right panel), supporting that the mobility shift was driven by phosphorylation rather than an intrinsic change in protein size. In addition, the ERK inhibitor treatment did not apparently affect the ouabain-induced IS1 autolysis of CAPN3:S606L (Fig. 3*A*, arrowhead in the right upper panel), suggesting that ERK does not substantially affect IS1 autolytic processing of CAPN3:S606L under these conditions.

**Figure 3:**
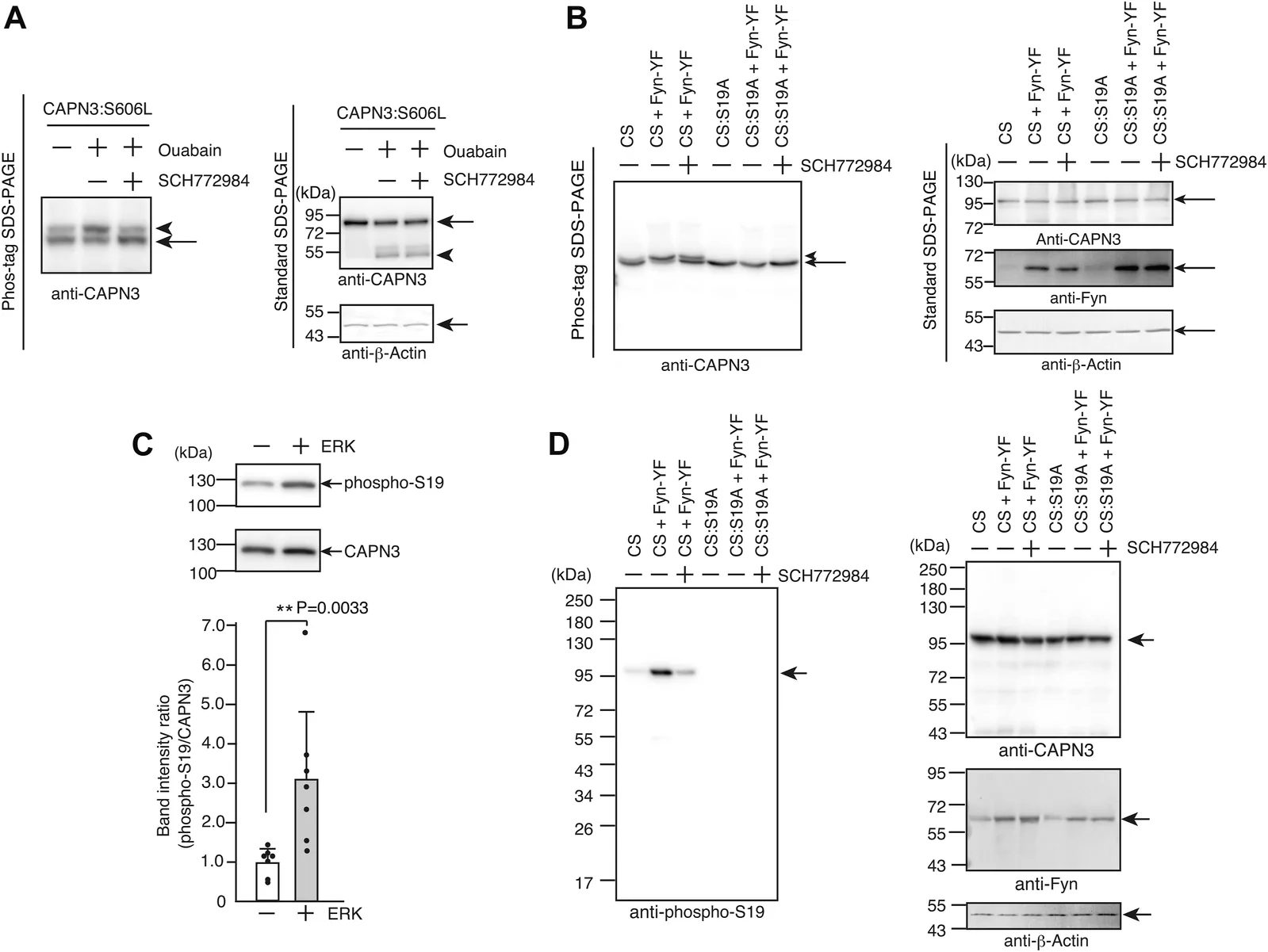
**ERK phosphorylates CAPN3 at serine 19.***A*, ERK inhibition reduces the ouabain-induced mobility shift of CAPN3:S606L. The ERK inhibitor SCH772984 (1 μM) decreases the phospho-shift in HeLa cell lysates in a Phos-tag SDS-PAGE (*left panel*). An *arrowhead* and *arrow* represent the phosphorylated and non-phosphorylated forms of CAPN3:S606L, respectively. N = 4. *Right panel* shows Western blot in a standard SDS-PAGE. N = 3. *Arrowhead* indicates autolytic ∼55 kDa band of CAPN3:S606L by ouabain. *B*, serine 19 is essential for the Fyn-YF-induced mobility shift of CAPN3:CS. The S19A mutant failed to display the phosphorylation-dependent mobility shift in HeLa cell lysates on a Phos-tag SDS-PAGE gel (*left panel*). N = 3. Right panel shows Western blot on a standard SDS-PAGE. *C*, ERK directly phosphorylates MBP-tagged CAPN3:CS *in vitro*. MBP-CAPN3:CS transiently expressed in HEK293T was purified and incubated with recombinant ERK *in vitro*. Representative western blots (*upper panels*) probed with anti-phospho-S19 and anti-CAPN3 antibodies are shown, while the *lower panel* displays the quantification of the phospho-S19/total CAPN3 intensity ratio. ERK (−): 1.0 ± 0.359; ERK (+): 3.14 ± 1.75 (mean ± SD). N = 7, ∗∗*p* = 0.0033, *Mann Whitney U test*. *D*, Fyn-YF induces the phosphorylation of CAPN3 at S19 in HeLa cells. The *left panel* shows a Western blot probed with an anti-phospho-S19 antibody; the *rightupper* and *lower panels* show blots for confirming expression levels of CAPN3, Fyn, and β-Actin, respectively. N = 3. For ERK inhibition, transfected cells were treated with 2.5 μM SCH772984 for 1 h prior to cell lysis.

To determine whether ERK directly phosphorylates CAPN3, we performed an *in vitro* kinase assay using immunopurified MBP-CAPN3:CS from HEK293T cells as the substrate. As shown in Figure 3*C*, incubation of MBP-CAPN3:CS with recombinant ERK in the presence of ATP greatly increased the signals detected with our newly developed anti-phospho-S19-specific antibody (Fig. 3*C*, upper panel) by approximately threefold (Fig. 3*C*, lower panel). These results demonstrated that ERK directly phosphorylates CAPN3 at S19 *in vitro* and in living cells.

To confirm the phosphorylation of the CAPN3 S19 residue, we substituted S19 with alanine (CAPN3:CS:S19A) and examined whether this mutation abolished the Fyn-YF-induced mobility shift in the Phos-tag SDS-PAGE (Fig. 3*B*). This mutant was almost exclusively detected in the lower band position, and a mobility shift was not observed even in the presence of Fyn-YF co-expression, indicating that the S19A mutation abolished the phosphorylation of CAPN3:CS that was detected in our Phos-tag SDS-PAGE assay (Fig. 3*B*, left panel).

We also demonstrated that the co-expression of Fyn-YF significantly increased the phospho-S19 signals of CAPN3:CS, which were inhibited by an ERK inhibitor (Fig. 3*D*, left panel). No signals were detected for CAPN3:CS:S19A using this antibody, thus validating its specificity (Fig. 3*D*, anti-phospho-S19 *versus* anti-CAPN3). These results suggested that ERK phosphorylates CAPN3 at the S19 residue in living cells.

### The phosphorylation of S19 did not affect the subcellular localization of CAPN3 in the skeletal muscle of mice

We previously reported that both endogenous CAPN3 and exogenous EGFP-CAPN3 delivered by an adeno-associated virus (AAV) predominantly localize to the sarcomere M-band in the skeletal muscle of mice 15, 26, 29. To analyze the effect of the phosphorylation of CAPN3 S19 on its subcellular localization *in vivo*, we constructed AAV vectors encoding EGFP-CAPN3:S19A and the phosphomimetic mutant, EGFP-CAPN3:S19D, and injected into the tibialis anterior (TA) of CAPN3-deficient (*Capn3*^−/−^) mice. Four weeks after AAV injection, we examined their localization in the skeletal muscle (Fig. 4) and detected EGFP-CAPN3 in the M bands of TA muscle sarcomeres. As for EGFP-CAPN3:S19A and EGFP-CAPN3:S19D, no apparent change in their localization was observed; both CAPN3:S19A and CAPN3:S19D were predominantly localized at the M-bands of sarcomeres of skeletal muscle as in the case of WT CAPN3 (Fig. 4). Thus, we concluded that the phosphorylation of S19 did not significantly affect the subcellular localization of CAPN3 *in vivo*.

**Figure 4:**
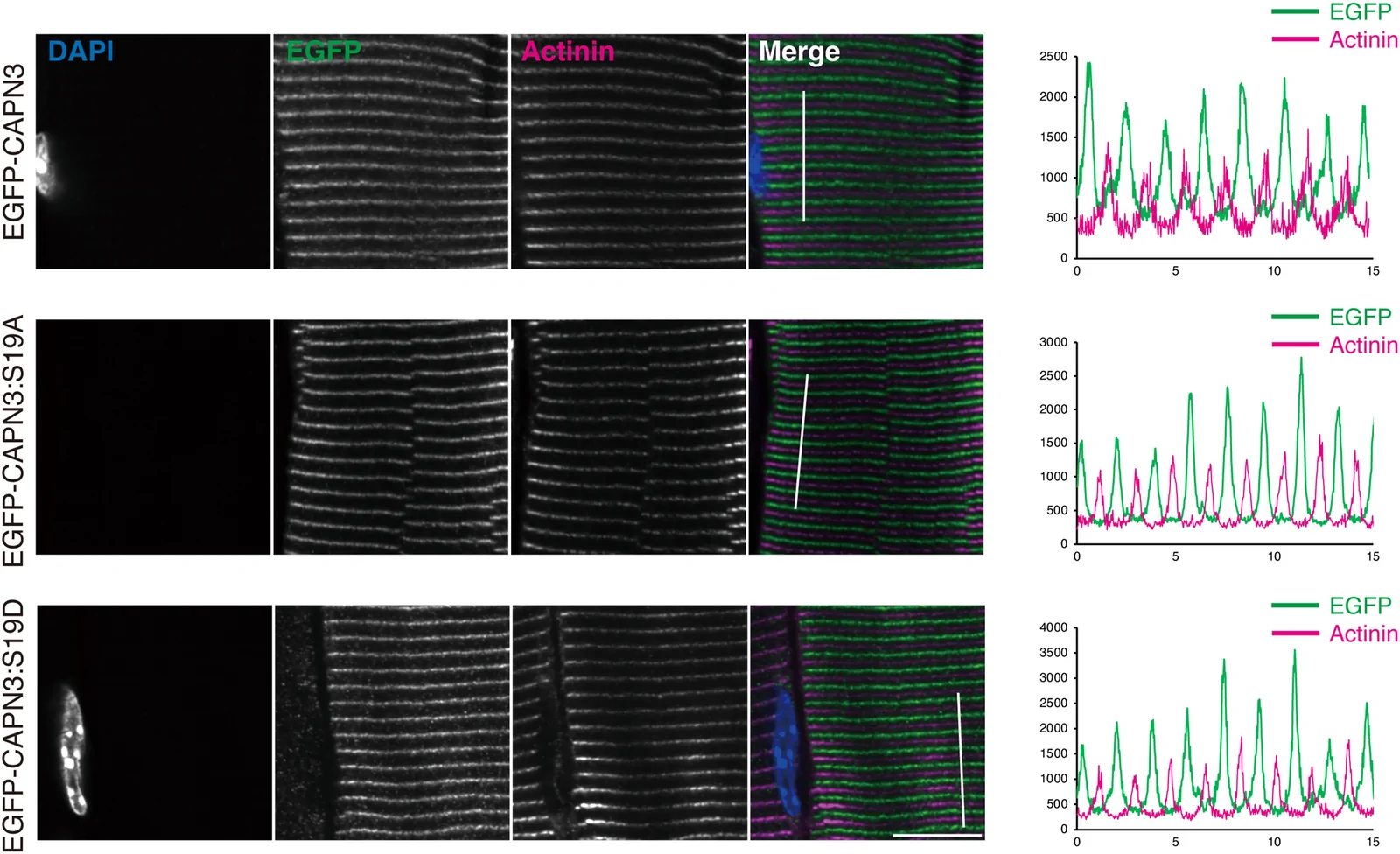
**Subcellular localization of S19 mutants delivered by AAV in the skeletal muscle of mice.** AAV vectors encoding EGFP-CAPN3 or its S19A and S19D variants were used to infect the tibialis anterior muscle of *Capn3*^−/−^ mice. After 4 weeks, muscle sections were harvested and stained with anti-α-actinin (*magenta*) and GFP (*green*) antibodies. The *right panels* show the intensity plots of immunostaining along the line in each merged image, with distance expressed in micrometers (µm) on the x-axis. For each AAV construct, both tibialis anterior muscles of two *Capn3*^−/−^ mice were injected and analyzed, and representative data are shown.

### S19 phosphorylation selectively modulates N-terminal processing of CAPN3

To further evaluate the impact of S19 phosphorylation on autolytic processing of CAPN3, we compared WT CAPN3 with the phosphorylation-deficient S19A and the phosphomimetic S19D mutants in HEK293T cells. IS1 processing was examined with a CAPN3 antibody (Fig. 5*A*), while N-terminal processing was examined with an AIS1 antibody that we developed in-house (Figs. 1*A* and 5*B*) (15). IS1 processing was not altered in CAPN3:S19 mutants compared with WT CAPN3 (Fig. 5*A*). In contrast, conversion of band a to band b, representing N-terminal processing, was reduced in the S19A mutant (Fig. 5*B*, left panel and Fig. 1*A*). Quantification of the intensity ratio b/a revealed that N-terminal processing was significantly decreased in CAPN3:S19A compared to that in WT CAPN3 and in the phosphomimetic mutant CAPN3:S19D (Fig. 5*B*, right panel). These results indicate that S19 phosphorylation selectively modulates N-terminal processing of CAPN3 without substantially affecting IS1 autolytic processing (Fig. 5*A*).

**Figure 5:**
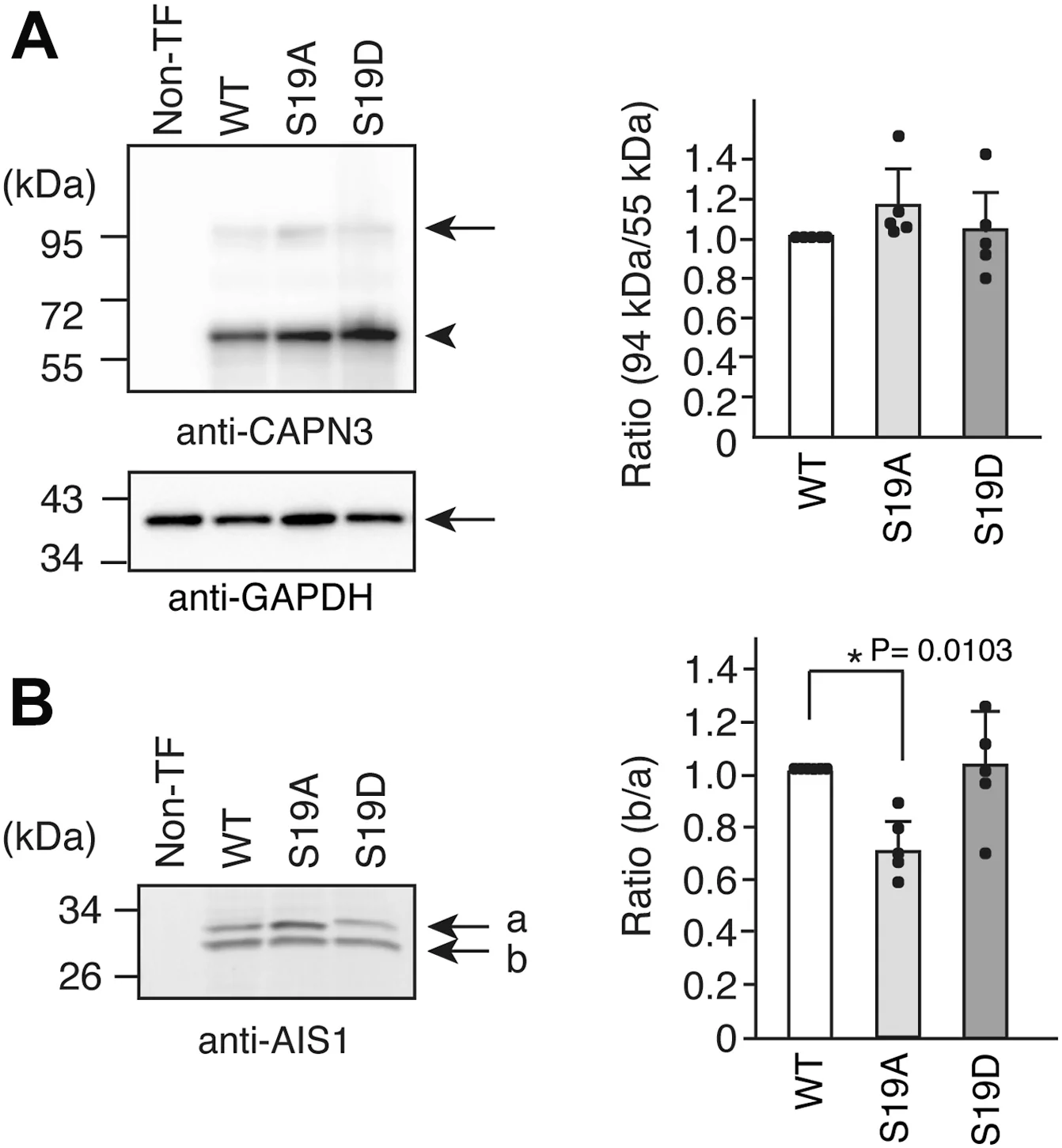
**Autolytic processing of CAPN3 S19 phosphorylation site mutants.***A*, Western blotting of the cell lysate expressing wild-type CAPN3 and CAPN3:S19 mutants. *Left panel*: Western blot probed with anti-CAPN3 antibody. An *arrow* and *arrowhead* indicate full-length of CAPN3 (*arrow*) and its autolyzed 55 kDa fragment (*arrowhead*), respectively. A representative image is shown. *Right panel*: quantification of the intensity ratio of the 94 kDa band (*arrow*) to the 55 kDa band (*arrowhead*). N = 5. S19A: 1.151 ± 0.182; S19D: 1.031 ± 0.209 (mean ± SD, data were normalized to the value of WT). *B*, the same set of cell lysate was analyzed using an anti-AIS1 antibody. The *left panel* shows a representative Western blot probed with anti-AIS1 antibody. Fragments a and b are depicted in Figure 1*A*. The *right panel* shows the quantification of the intensity ratio of the fragment b to the fragment a. S19A: 0.717 ± 0.108; S19D: 1.035 ± 0.189 (mean ± SD, data were normalized to the value of WT). N = 5, ⁎*p* = 0.0103, *Steel's multiple comparison test*.

### IGF-1 increases the S19 phosphorylation of CAPN3 in cultured skeletal muscle cells

ERK is activated downstream of numerous extracellular signals, including hormones and growth factors. We therefore investigated whether S19 phosphorylation of CAPN3 is induced by IGF-1, a key regulator of skeletal muscle growth and regeneration. For this experiment, we used cultured myotubes derived from CAPN3KI mice expressing CAPN3:CS, since the mutant protein is stable and expressed at higher levels than WT CAPN3, enabling a more sensitive detection of CAPN3 phosphorylation, as exemplified by the case of ouabain treatment (Fig. 1*D*). IGF-1 treatment resulted in a marked increase in S19 phosphorylation of CAPN3 (Fig. 6). These data suggested that S19 phosphorylation can be induced by a physiologically relevant extracellular signal in skeletal muscle cells and represents the linkage of IGF-1 signaling to CAPN3.

**Figure 6:**
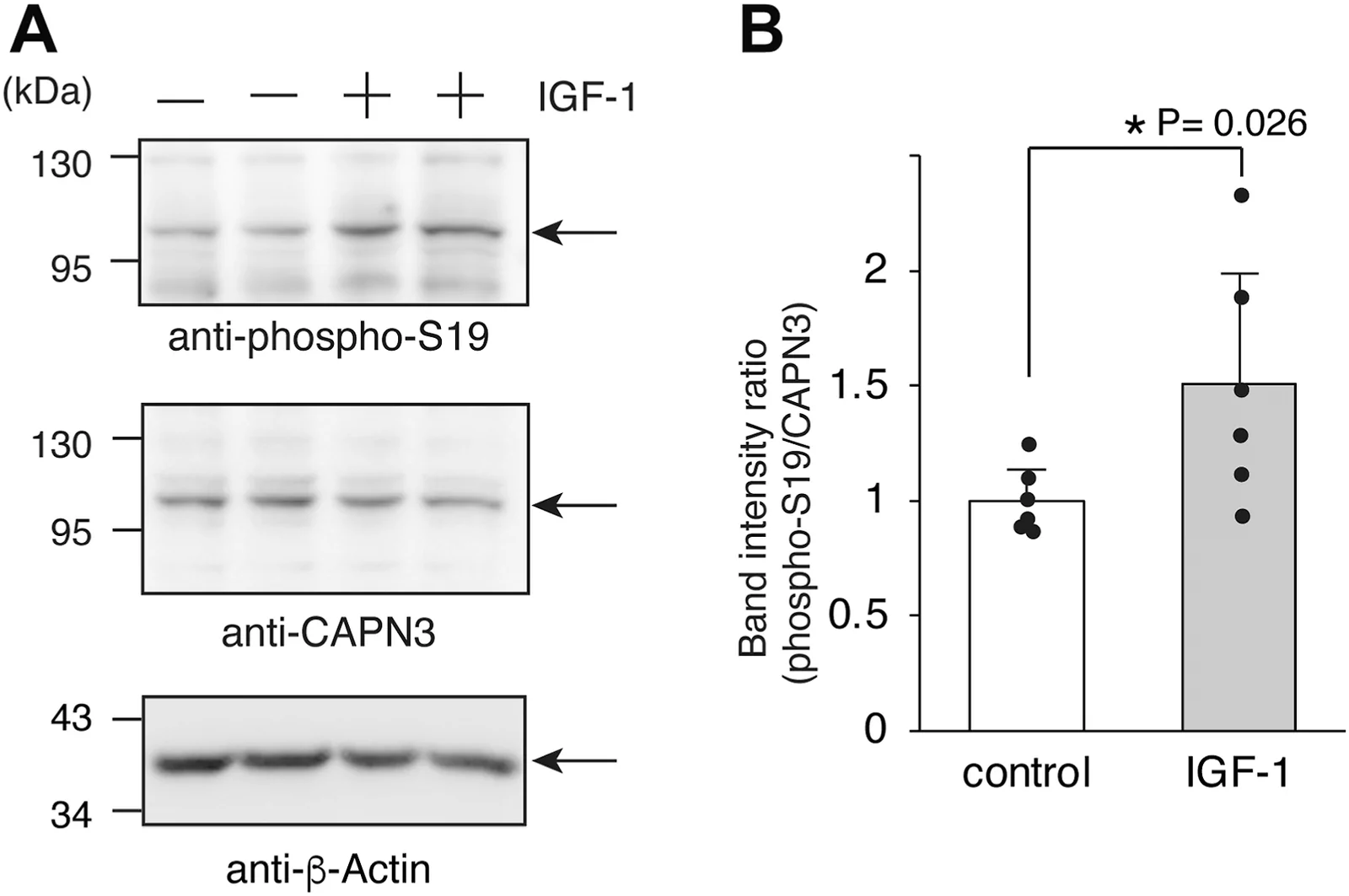
**IGF-1 induces S19 phosphorylation of CAPN3:CS in skeletal muscle cells.***A*, after 6 days of differentiation, CAPN3KI muscle cells were treated with IGF-1 (100 ng/ml) for 15 min. The cell lysates were separated on a 7.5% SDS-PAGE gel and were subjected to western blotting and then probed with anti-phospho-S19 (*upper panel*), anti-CAPN3 (*middle panel*), and anti-β−Actin (*lower panel*) antibodies. Representative images are shown. N = 6. *B*, band intensity ratio of phospho-S19/CAPN3. Data are shown as mean ± SD. Control: 1.0 ± 0.132; IGF-1: 1.50 ± 0.482 (mean ± SD). N = 6, ⁎*p* = 0.026, *Mann-Whitney U test*.

## Discussion

Ouabain is an ideal chemical compound for effectively activating CAPN3 in both heterologous cells and cultured mouse myotubes 15, 25. This triggers a subtle but long-lasting cytoplasmic increase in Ca^2+^ levels, which is essential for CAPN3 activation (15). In this study, we identified phosphorylation of CAPN3 upon ouabain-induced CAPN3 activation. We found that ouabain induced the ERK-mediated phosphorylation of CAPN3 at S19 within the NS region, and that recombinant ERK directly phosphorylated S19 *in vitro*. Importantly, the phosphorylation-deficient S19A mutation reduced N-terminal processing of CAPN3 without substantially affecting IS1 autolytic processing (Fig. 5). Neither S19A nor the phosphomimetic S19D mutation caused an apparent change in the localization of CAPN3 at the sarcomeric M-band (Fig. 4). Moreover, we found that S19 phosphorylation is induced by IGF-1, a key regulator of muscle growth and regeneration (Fig. 6). Together, these findings identify ERK-mediated phosphorylation as a previously unknown mechanism that selectively modulates N-terminal processing of CAPN3.

Phosphorylation-dependent regulation of protease activity has been reported for several members of the calpain family 18, 19, 20, 21, 30. Shiraha *et al*. reported that protein kinase A-mediated phosphorylation of m-calpain (calpain-2) by epidermal growth factor limits its activation (18). It has also been reported that m-calpain activation is induced by the mitogen-activated protein kinase (MAPK)-mediated phosphorylation by epidermal growth factor (19) and brain-derived neurotrophic factor (30). Activation of the ERK pathway by extracellular signals results in calpain B phosphorylation and activation in fruit flies (21). Although the mechanism of calpain activation *in vivo* remains a subject of ongoing debate (8), our findings extend the role of phosphorylation in the calpain family by demonstrating that ERK-dependent phosphorylation of CAPN3 regulates a specific autolytic processing event rather than measurably altering its overall proteolytic activation.

The distinction between N-terminal processing and IS1 autolysis is important for interpreting the effect of S19 phosphorylation. The S19A mutation significantly decreased conversion of fragment a to fragment b (Figs. 1*A* and 5), indicating that phosphorylation facilitates N-terminal processing. In contrast, IS1 processing was not substantially altered by the S19A mutation. Because removal of IS1 is a critical step required for full CAPN3 proteolytic activity 13, 14, we consider that S19 phosphorylation does not increase the intrinsic protease activity of CAPN3. Instead, S19 phosphorylation appears to selectively regulate processing of its N-terminal region.

The functional consequence of this selective N-terminal processing remains to be elucidated. In CAPN1 and CAPN2, removal of the N-terminal region has been associated with altered Ca^2+^ sensitivity 9, 31, 32, 33, 34, raising the possibility that N-terminal processing of CAPN3 may similarly influence its enzymatic properties. However, this possibility is difficult to assess under the experimental conditions used here; surrogate indicators of activation other than IS1 processing need to be established. Likewise, because the structure of the CAPN3 N-terminal region has not been resolved, whether S19 phosphorylation alters its local conformation, susceptibility to autolytic cleavage, and/or interaction with other proteins remains unknown. Further structural and biochemical studies are required to address these questions.

A limitation of this study is the absence of a reliable *in vitro* method to quantitatively measure the proteolytic activity of the intact full-length form of CAPN3 and its S19 mutants under precisely controlled Ca^2+^ conditions or examine subtle effects of S19 phosphorylation on other biochemical properties of CAPN3. Nevertheless, under the experimental conditions examined here, S19 phosphorylation clearly affected N-terminal processing without a detectable alteration in IS1 processing.

S19 phosphorylation was also induced by IGF-1 in cultured skeletal muscle cells. IGF-1 activates both the Ras/Raf/MEK/ERK and PI3K/Akt/mTOR pathways and plays important roles in skeletal muscle growth and regeneration 35, 36. The ability of IGF-1 to induce S19 phosphorylation in cultured skeletal muscle cells therefore indicates that CAPN3 phosphorylation is not only induced by pharmacological stimulation like ouabain, but can occur downstream of physiologically relevant extracellular signals triggering ERK pathway 37, 38, 39. Determining when and where S19 phosphorylation occurs in skeletal muscle *in vivo*, and whether it changes in response to muscle contraction, injury, or regeneration, will be important subjects for future investigation.

In summary, our study identifies S19 as an ERK-dependent phosphorylation site in CAPN3 and demonstrates that this phosphorylation selectively modulates N-terminal processing without substantially altering IS1 autolysis. These findings reveal that distinct autolytic processing events within CAPN3 can be differentially regulated and provide a basis for investigating the physiological significance of N-terminal processing in skeletal muscle.

## Experimental procedures

### Construction of expression vectors

Expression vectors for wild-type and mutants of CAPN3, in the form of pSRD-human CAPN3 and pAAV-CMV-EGFP-CAPN3, have been described in our previous papers 29, 40. Mutations were introduced by site-directed mutagenesis with the following primers: hCal3 S19A-sense, cggctgagccccggGccccagggccagttcctc; hCal3 S19A-antisense, gaggaactggccctggggCccggggctcagccg; hCal3 S19D-sense, cggctgagccccggGAcccagggccagttcctc; hCal3 S19D-antisense, gaggaactggccctgggTCccggggctcagccg. Deletion mutants of CAPN3 have been described previously (41). To construct the pAAV-CMV-maltose-binding protein (MBP)-CAPN3:CS, an MBP fragment was amplified by PCR using the primers His-MalE-S: GTCAGATCCGCTAGCGCCACCatgaaaatccaccatcacc and His-MalE-AS: agCTCGAGATCTGAGTCCcatctggaagtacaggttctccc; the pMal-c6T vector (New England Biolabs Japan Inc. Tokyo, Japan) was used as the template. The amplified fragment was digested with *Nhe*I and *Xho*I and inserted into the *Nhe*I-*Xho*I site of pAAV-CMV-EGFP-CAPN3:CS to replace EGFP with MBP. Sequences were verified using the SupreDye v3.1 Cycle Sequencing Kit (M&S Techno Systems).

### Cell culture and transfection

HEK293T and HeLa cells were cultured in Dulbecco’s modified Eagle Medium (DMEM; Nacalai Tesque, 08458-16) supplemented with 10% fetal bovine serum, 100 units/ml of penicillin, and 100 μg/ml of streptomycin (Nacalai Tesque, Kyoto, Japan, 09367-34). Approximately 5 × 10^5^ cells in a 6 cm dish were transfected with 2 μg of the appropriate plasmid using 6 μl of PEI-MAX 40k (Polysciences Inc., Warrington, PA, USA). After 20 to 24 h, the cells were lysed with a lysis buffer [10 mM Tris-HCl (pH 7.0), 150 mM KCl, 1 mM EGTA-KOH (pH 8.0), and 1.0% Triton] supplemented with cOmplete Protease Inhibitor Cocktail (Sigma-Aldrich, 04693116001) for 10 min on ice. To examine the effect of the ERK inhibitor on ouabain treatment, cells were preincubated with 1 μM SCH772984 for 15 min, followed by treatment with 1 mM ouabain for 60 min in the presence of the inhibitor. To assess the effect of ERK inhibition on Fyn-YF–mediated CAPN3 phosphorylation, transfected cells were treated with SCH772984 (2.5 μM) for 60 min before harvesting. The lysates were centrifuged at 20,630*g* for 10 min, and the resulting supernatants were mixed with 4 × SDS-PAGE sample buffer and boiled at 95 °C for 5 min. For the Phos-tag SDS-PAGE, MnCl_2_ (final concentration 3 mM) was added to the sample.

Myoblasts from WT and CAPN3KI mice were obtained and cultured as described previously (15). For differentiation, the cells (at a density of ∼ 2.0 × 10^5^) in proliferation medium (PM) were plated overnight in 3.5 cm dishes coated with 20 times diluted Matrigel. The next day, the medium was changed to the differentiation medium (DMEM containing 5% horse serum and penicillin-streptomycin). To examine IGF-1-induced CAPN3 phosphorylation, cells were treated with 100 ng/ml of IGF-1 for 15 min on day 6 after differentiation. After IGF-1 stimulation, the cells were washed with ice-cold PBS and quickly lysed with 350 μl of lysis buffer supplemented with cOmplete Protease Inhibitor Cocktail, 100 μM Na_3_VO_4_, and PhosSTOP (Merck, 4906845001) at 4 °C. The lysates were centrifuged at 20,630*g* for 10 min at 4 °C, and the resulting supernatants were mixed with 4 × SDS-PAGE sample buffer and boiled at 95 °C for 5 min.

### Western blotting

The samples were separated using an Extra PAGE One Precast Gel 7.5 to 15% (Nacalai Tesque, 13065–54) except Figure 6 (7.5% SDS-PAGE gel) and transferred to a membrane, which was blocked with 5% skim milk/PBST or 5% BSA/PBST for 1 h at room temperature (RT). After blocking, the membrane was probed with the appropriate primary antibody for 1 h at RT. After washing three times with PBST, the membrane was probed with horseradish peroxidase (HRP)- or Alkaline phosphatase (AP)-conjugated secondary antibodies for 1 h at RT. Following another three washes with PBST, the protein bands were detected using Immobilon Western Chemiluminescent HRP Substrate (Millipore) and visualized using LAS3000 (Fujifilm, Tokyo, Japan). For detection of bands recognized with AP-conjugated secondary antibodies, Western Blue Stabilized Substrate for Alkaline phosphatase (Promega, Tokyo, Japan, S3841) was used. The band intensities were analyzed using Fiji open-source software (42).

For the Phos-tag SDS-PAGE, the samples were separated on a 6.75% SDS-PAGE gel containing 33 μM Phos-tag (NARD Institute, Ltd., AAL-107), and 66 μM MnCl_2_. The gel was then washed three times with a transfer buffer containing 10 mM EDTA for 30 min. After an additional 10-min rinse in transfer buffer, the proteins were transferred to a membrane, and immunoblotting was performed as described earlier.

### Antibodies

The antibodies used in this study were rabbit anti-CAPN3 polyclonal antibody (1 μg/ml for western blotting; 2 μg/ml for immunohistochemistry; Proteintech Japan, Tokyo, Japan, 28476-1-AP), anti-α-actinin antibody (2 μg/ml for immunohistochemistry; Sigma-Aldrich, EA-53), anti-GFP polyclonal antibody (2 μg/ml for immunohistochemistry; MBL, Tokyo, Japan, Code No. 598), and anti-β-Actin antibody (1 μg/ml for Western blotting; MBL, Code No. M177-3), anti-Fyn antibody (1 μg/ml for western blotting; Proteintech, 66606-1-Ig), and anti-GAPDH antibody (1 μg/ml for Western blotting; Proteintech, 10494-1-AP). The AIS1 antibody was described in our previous study (15) and used at 1 μg/ml for western blotting. The production of rabbit anti-phosphoS19 antibodies using a KLH-conjugated synthetic peptide containing phosphorylated-S19 (pS19) [CGAEPR(pS)PGPVP] was performed by SCRUM, Inc. and used at 1 μg/ml for western blotting.

### *In vitro* kinase assay

HEK293T cells were transfected with the pAAV-CMV-MBP-CAPN3:CS vector using PEI-Max. After 36 h, cells were lysed on ice for 10 min with lysis buffer containing 10 mM Tris-HCl (pH 7.0), 150 mM KCl, 1 mM EGTA-KOH (pH 8.0), and 1.0% Triton, supplemented with cOmplete Protease Inhibitor Cocktail (Sigma-Aldrich, 04693116001). Cell lysates were centrifuged at 20,630*g* for 10 min. To the supernatants, 30 μl of a 50% amylose-resin slurry was added and rotated for 1 h at 4 °C. The resin was washed twice with lysis buffer and then twice with kinase buffer [25 mM Tris-HCl (pH 7.5), 1 mM EGTA-KOH (pH 8.0), and 10 mM MgCl_2_], and resuspended in 30 μl of the same buffer supplemented with 1 mM ATP and 100 μM Na_3_VO_4_. For the kinase assay, 0.1 μg of active ERK1 (Merck, 14-439-M) was added to 10 μl of the MBP-CAPN3:CS preparation and incubated at 30 °C for 15 min. The reaction was stopped by adding 4 × SDS-PAGE sample buffer, and the samples were boiled for 5 min at 95 °C.

### Production and infection of AAV

AAV encoding EGFP-CAPN3 and its derivatives were produced as previously described (29). Briefly, HEK293T cells in a collagen-coated 225 cm^2^ culture flask (AGC Techno Glass Co. Ltd, Shizuoka, Japan, IWAKI 4143-010) were transfected with three plasmids—pHelper vector (AAVpro Helper Free System, Takara), pAAV2/9n (Addgene, #112865), and pAAV-CMV-EGFP-CAPN3 or its derivatives—via PEI-MAX-mediated transfection. After 4 h, the media was replaced with 35 ml of DMEM without fetal bovine serum, and the cells were cultured for an additional 5 days in a CO_2_ incubator at 37 °C, without changing the medium.

The culture medium containing AAV was centrifuged at 830*g* for 15 min at RT. The resulting supernatant was treated with 8.5 ml of AAVanced Concentration Reagent (System Biosciences, San Jose, CA, USA, AAV100A-1) or 40% polyethylene glycol in a 2.5 M NaCl solution. After 3 days of incubation at 4 °C, the solution was centrifuged at 830*g* for 30 min. The pellet was washed with 1 ml cold PBS, centrifuged again at 830*g* for 1 min, and then resuspended in 350 μl cold PBS. The AAV solution was aliquoted and stored at −80 °C until use.

The experimental animals were handled in accordance with the guidelines of the Experimental Animal Care and Use Committee of the Tokyo Metropolitan Institute of Medical Science (approval number: 24-034). The detailed procedure was described in our previous study (29). For *in vivo* delivery, AAV (5 × 10^10^ genome copies per muscle) was injected into the TA muscle of anesthetized *Capn3*^−/−^ mice (5 months old) with isoflurane using 27G insulin syringes (Terumo, Tokyo, Japan). After 4 weeks, the injected TA muscles were harvested, dissected, and processed for immunohistochemistry.

### Immunohistochemistry

TA skeletal muscles were immersed and fixed in 4.0% paraformaldehyde in PBS for 3 h at 4 °C. The frozen sections were cut into 10-micron-thick sections using cryostat CM1950 (Leica Biosystems). The sections were mounted on glass slides, permeabilized with PBS containing 0.2% Triton for 5 min at RT, and subjected to antigen retrieval using boiled 10 mM Tris–HCl (pH 9.0) for 10 min. After blocking in PBS containing 5.0% bovine serum albumin for 1 h at RT, the sections were probed with anti-GFP (Code No. 598; 1:500) and anti-actinin (EA-53; 1:500) antibodies overnight at 4 °C. After three washes with PBS, the sections were probed with Alexa Fluor 488 goat anti-rabbit IgG (H + L) cross-adsorbed secondary antibody and Alexa Fluor 594 conjugated anti-mouse IgG antibody (Thermo Fisher Scientific, A-11034 and A11032, 1:1000) for 30 min at RT. After washing with PBS, the sections were mounted using Vibrance Antifade Mounting Medium with 4′,6-diamidino-2-phenylindole (Vector Laboratories, Inc., H-1800) and observed under a confocal fluorescence microscope (FV3000, Evident Corporation).

### Determination of the CAPN3 phosphorylation site using mass spectrometric analysis

HEK293T cells grown in 10 cm dishes were transfected with 2 μg of pAAV-CMV-MBP-CAPN3:CS, either alone or in combination with 2 μg of the pME-Fyn-YF vector (27). After 24 h of transfection, the cells were lysed in lysis buffer supplemented with cOmplete Protease Inhibitor Cocktail and PhosSTOP (Merck, 4906845001) at 4 °C for 10 min. The lysates were clarified by centrifugation at 20,630*g* for 10 min. To the supernatant, 60 μl of a 50% amylose-resin slurry was added and rotated for 2 h at 4 °C. The MBP-CAPN3:CS–bound resin was washed four times with lysis buffer and once with PBS. 50 μl of 2 × SDS-PAGE sample buffer containing 3 mM MnCl_2_ were then added to the samples, and the mixture was boiled for 5 min.

The samples were separated on a 6.75% SDS-PAGE gel containing 33 μM Phos-tag and 66 μM MnCl_2_ and stained with Oriole Gel Stain (Bio-Rad Laboratories, Inc, #1610495). Because the phosphorylated and non-phosphorylated MBP-CAPN3:CS bands were difficult to resolve, the entire band corresponding to MBP-CAPN3:CS was excised from the gel with a scalpel.

Excised gels were digested with trypsin (Promega, Madison, WI, USA, V5111) (43). LC-MS/MS analyses were performed in data-dependent acquisition (DDA) mode using an Easynano1200 (Thermo Fisher Scientific) equipped with a C18 capillary column (0.075 mm I.D. × 100 mm L, Nikkyo Technos) and Triple TOF 5600+ (Sciex). Peptides were eluted at a flow rate of 300 nl/min with a linear gradient from 0% to 50% mobile phase B over [73/113] min, followed by a ramp to 95% B for 1 min, a hold at 95% B for 5 min, and re-equilibration at 0% B for 5 min (total run time: [85/125] min). Mobile phase A consisted of 0.1% formic acid in water, and mobile phase B consisted of 0.1% formic acid in 80% acetonitrile/20% water. Tandem mass spectra were acquired with Analyst TF software (version 1.2.0.3, Sciex). MS parameters are described in Table S2 of supporting information.

The raw mass spectrometric data were analyzed with ProteinPilot version 5.0.1 software (Sciex) using the Paragon algorithm with “Gel-Based ID” and “Biological modifications” setting against the UniProt 2010.06.22 database (released June 2010, 35115 entries) supplemented with 262 frequently observed contaminants, including human keratins, bovine serum proteins, and proteases. Precursor and product ion mass tolerances were 0.05 and 0.1 Da, respectively and up to one missed cleavage was allowed. The mass spectrometry proteomics data were deposited in the ProteomeXchange Consortium *via* the jPOSTrepo as PXD076638 (44).

Among the peptides detected with > 95% confidence, those derived from CAPN3 were subjected to further analyses. Peptides with the same phosphorylated sites and non-phosphorylated counterparts were grouped together, respectively. Data were obtained from two independent experiments, with two technical replicate measurements performed for each experiment, and the numbers of detections were averaged (Table S1).

### Statistical analysis

Statistical analyses were performed using the EZR software (45). All data are presented as mean ± standard deviation, and individual data points are shown as scatter plots in the figures.

## Data availability

The data supporting the findings of this study are available within the article and the supporting information. The mass spectrometry proteomics data were deposited in the ProteomeXchange Consortium *via* the jPOSTrepo as PXD076638.

## Supporting information

This article contains supporting information.

## Conflict of interest

The authors declare that they have no conflicts of interest regarding the content of this article.
