# Supplementary material for: ERK-mediated phosphorylation modulates the autolytic processing of the cysteine protease CAPN3
Source: J Biol Chem. 2026 Oct 14;302(12):113638. doi: 10.1016/j.jbc.2026.113638 (PMC13769584; doi:10.1016/j.jbc.2026.113638)
Supplement: Supplementary file 1 [file mmc1.pdf]

Supporting information

Title

ERK-mediated phosphorylation modulates the autolytic processing of the cysteine protease CAPN3

Authors

Chihiro Hisatsune, Fumiko Shinkai-Ouchi, Mayumi Shindo, Shoji Hata, and Yasuko Ono

Contents

Tables S1 and S2

		CS			CS+FynYF		
		detection		ratio	detection		ratio
site	sequence	Total	phospho-	(%)	Total	phospho-	(%)
S19	TAAEPR**S**PGPVPHPAQSK<br>**S**PGPVPHPAQSK	100.0	15.3	19.7	46.5	20.0	43.5
T184	YGEWVDVVIDDCLP**T**YNNQLVFTK	115.8	0.8	0.7	81.5	0.5	0.4
S265	GSLMGC**S**IDDGTNMTYGTSPSGLNMGELIAR	158.0	0.0	0.0	136.3	0.8	0.6
S400	LQHQVTEDGEFWM**S**YEDFIYHFTK	132.3	0.3	0.2	99.3	0.0	0.0
S423	LEICNLTADALQ**S**DKLQTWTVSVNEGR	63.0	0.8	1.1	61.3	0.0	0.0
S474	LKLLEEDDDPDD**S**EVICSFLVALMQK<br>LLEEDDDPDD**S**EVICSFLVALMQK	178.5	1.3	0.7	94.0	0.3	0.1
S611	AN**S**NKELGVDQESEEGKGKTSPDKQK<br>AN**S**NKELGVDQESEEGKGK	21.8	0.0	0.0	30.8	0.8	2.7
S621	ANSNKELGVDQE**S**EEGKGK<br>ANSNKELGVDQE**S**EEGKGKTSPDKQK<br>ELGVDQE**S**EEGKGKTSPDKQK	27.5	1.5	5.7	36.3	2.3	5.6
T628	ANSNKELGVDQESEEGKGK**T**SPDKQK<br>ELGVDQESEEGKGK**T**SPDKQK	8.5	0.3	5.0	10.3	0.5	8.3
S629	ANSNKELGVDQESEEGKGKT**S**PDKQK<br>ELGVDQESEEGKGKT**S**PDKQK	9.0	5.5	61.7	10.8	7.0	66.1
S636	TSPDKQKQ**S**PQPQPGSSDQESEEQQQFR<br>QKQ**S**PQPQPGSSDQESEEQQQFR<br>Q**S**PQPQPGSSDQESEEQQQFR	48.8	0.3	0.7	56.8	2.0	3.4
S643	TSPDKQKQSPQPQPG**S**SDQESEEQQQFR<br>QKQSPQPQPG**S**SDQESEEQQQFR<br>QSPQPQPG**S**SDQESEEQQQFR	48.8	0.3	0.5	56.8	0.3	0.4
S644	TSPDKQKQSPQPQPGS**S**DQESEEQQQFR<br>QKQSPQPQPGS**S**DQESEEQQQFR<br>QSPQPQPGS**S**DQESEEQQQFR	48.8	1.0	1.9	56.8	1.0	1.8
T689	**T**HGFTLESCR	50.0	0.5	0.8	48.0	0.0	0.0
S696	THGFTLE**S**CR	50.0	0.5	1.3	48.0	0.3	1.3

Table S1. Summary of the phosphorylated CAPN3 sites detected in this study. The phosphorylated residues in each peptide sequence are shown in bold. The totals represent the non-phosphorus and phosphorus forms. For phosphorylated sites detected in more than one peptide sequence, the values for the corresponding peptides were summed per experiment. The average values from four experiments for peptide detection (confidence >95%) and the ratios of the phosphorylated forms are shown.

Parameter	MS1 (TOF MS)	MS2 (TOF MS²)
**General Settings**		
Experiment type	TOF MS	TOF MS² (IDA)
Polarity	Positive	Positive
Mass range (Da)	400–1250	100–1600
Accumulation time (ms)	250.1	100.0
Period cycle time (ms)	2301	2301
Resolution mode	High Resolution	High Resolution
**Ion Source Parameters**		
Source type	Nanospray	Nanospray
Curtain gas (CUR)	20	20
Ion source gas 1 (GS1)	20	20
Ion source gas 2 (GS2)	0	0
Interface heater temp (IHT, °C)	150	150
Ion spray voltage (ISVF, V)	2300	2300
**Fragmentation Parameters**		
Collision energy (CE, V)	10	35
Collision energy spread (CES, V)	—	±15
Collision type	—	CID (rolling CE)
Declustering potential (DP, V)	80	80
**IDA / Dynamic Exclusion Settings (MS2 only)**		
Charge state range	—	2–5
Minimum precursor mass (Da)	—	400
Minimum intensity threshold (counts)	—	125
Maximum MS/MS per cycle	—	20
Dynamic exclusion duration (sec)	—	12
Exclusion mass tolerance (mDa)	—	50
Exclude isotopes window (Da)	—	4
Maximum accumulation time (sec)	—	2

Table S2. MS acquisition parameters for the SCIEX TripleTOF 5600+

Abbreviations: CID, collision-induced dissociation; CUR, curtain gas; DP, declustering potential; GS1/GS2, ion source gas 1/2; IDA, information-dependent acquisition; IHT, interface heater temperature; ISVF, ion spray voltage floating; TOF, time-of-flight.
